# Botulinum Toxin and Redress in the United Kingdom, Findings From a Cross‐Sectional Survey

**DOI:** 10.1111/jocd.70677

**Published:** 2026-01-16

**Authors:** Lee Smith, José Francisco López‐Gil, Masoud Rahmati, Julia Gawronska, Roshan Ravindran

**Affiliations:** ^1^ Centre for Health, Performance and Wellbeing Anglia Ruskin University Cambridge UK; ^2^ Department of Public Health, Faculty of Medicine Biruni University Istanbul Turkey; ^3^ School of Medicine Universidad Espíritu Santo Samborondón Ecuador; ^4^ Vicerrectoría de Investigación y Postgrado Universidad de Los Lagos Osorno Chile; ^5^ CEReSS‐Health Service Research and Quality of Life Center, Assistance Publique‐Hôpitaux de Marseille Aix Marseille University Marseille France; ^6^ KLNIK The Colony Wilmslow Cheshire UK

Dear Editor,

In the United Kingdom, an estimated 900 000 cosmetic botulinum toxins (BoNT) injections are performed annually [1]. However, unlike other specialized medical interventions, in the United Kingdom many are delivered by individuals without formal healthcare qualifications and under minimal regulatory oversight. Owing to a lack of regulations in the United Kingdom surrounding BoNT, these injections can be carried out by practitioners with multiple backgrounds and levels of training.

A recent meta‐analysis of clinical trials found an overall BoNT complication rate of approximately 16% [2]. When a complication from BoNT occurs, it is good ethical practice for the practitioner to support the patient in finding a resolution. This may include correcting any errors where possible or referring the patient to other services such as NHS accidents and emergency (A&E) settings. Moreover, when a practitioner does not agree to support, the patient may have no other option than to attend such settings. In the United Kingdom, complications dealt with in A&E shifts care onto the National Health Service (NHS) resulting in significant costs [3].

An independent 2013 review of cosmetic interventions in the United Kingdom led by Sir Bruce Keogh concluded that “a person having a nonsurgical cosmetic intervention has no more protection and redress than someone buying a ballpoint pen or a toothbrush” [4]. However, despite this, to date, little has changed, and it is not known what level of redress is provided when patients experience complications, whether they are referred to A&E settings, and whether this level varies by practitioner type.

A cross‐sectional online survey gathered data on experiences with cosmetic BoNT injections across the United Kingdom. The survey was developed collaboratively by academic researchers and clinicians and was hosted online (using Jisc Online Surveys [London, England]) to maximize accessibility. The survey was launched in January 2025 and remained open to responses up to April 2025. Participation was open to adults (≥ 18 years) who had received cosmetic BoNT treatment with “medical doctors, dentists, nurses, beauticians, and other non‐healthcare practitioners.” Ethical approval was granted in December 2024 by the Anglia Ruskin University Research Ethics Panel (Ethics ID: ETH2425‐1930).

The survey included two questions in relation to redress: (1) Did the clinic or practitioner agree to support you to set things right. Response options were: Yes/No. (2) Did the clinic or practitioner advise you to go to an accident and emergency setting for any complications experienced after the procedure. Response options were: Yes/No. All survey questions were optional and thus participants did not need to complete all questions included in the survey. Given this approach the sample size varied for each survey question.

Categorical variables were summarized as numbers (*n*) and percentages (%). Differences in the distribution of responses across provider role categories were assessed using Fisher's exact test with Monte Carlo simulation (1 000 000 replicates), given that some contingency table cells had small expected counts. Statistical significance was defined as *p* < 0.05. All statistical analyses were conducted using R software (version 4.3.1; R Foundation for Statistical Computing, Vienna, Austria).

Among respondents, the proportion reporting that the clinic or practitioner agreed to support them to “set things right” ranged from 59.3% for beauticians to 81.2% for other non‐health care providers (Figure 1). Differences across provider roles were not statistically significant (Fisher's exact test, simulated *p* = 0.187).

**FIGURE 1:**
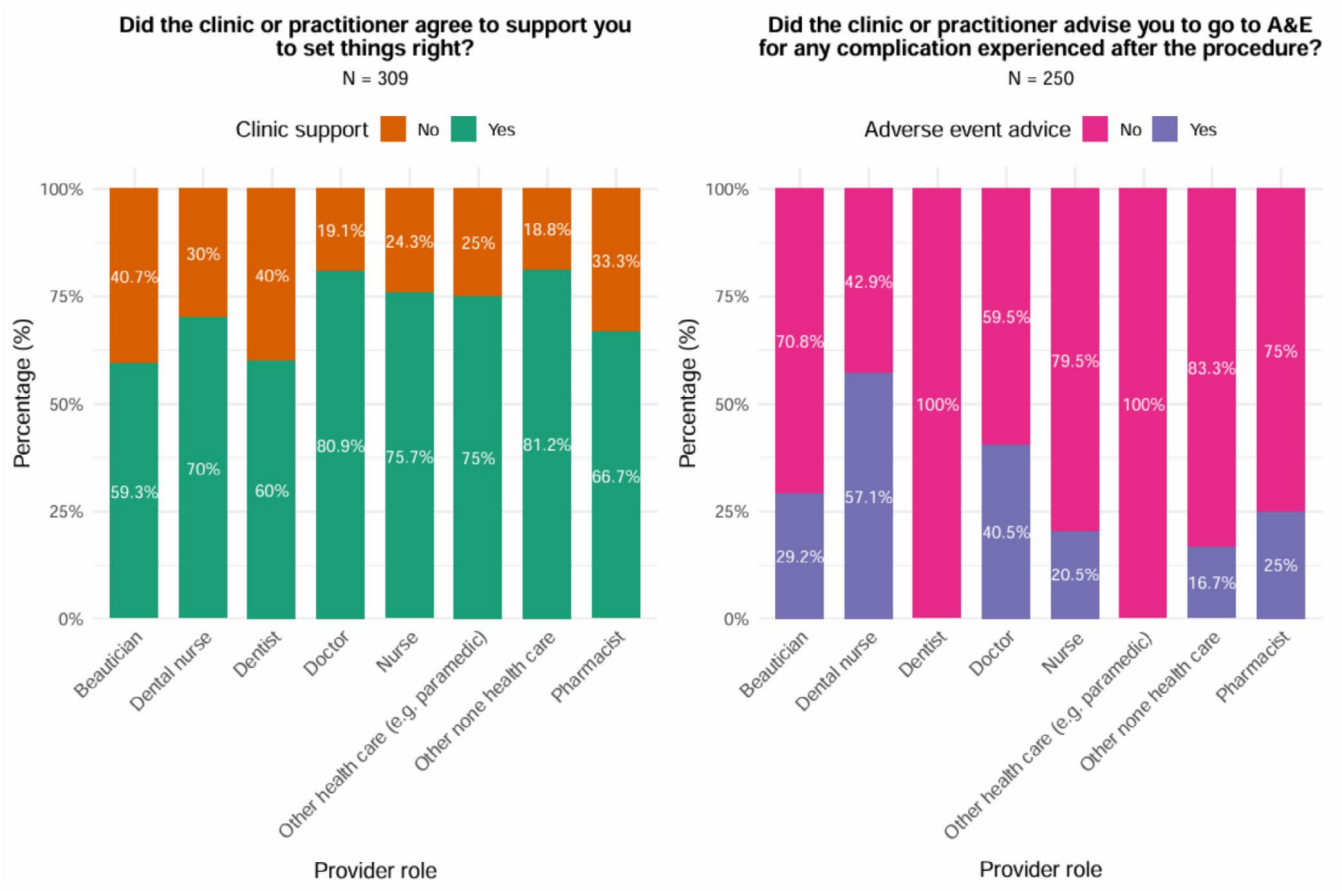
Responses on clinic/practitioner support and advice to attend A&E post‐procedure complications.

In contrast, the proportion of participants who reported being advised to attend the A&E department for any complication after the procedure varied substantially across provider roles, from 0% among dentists and other health care providers (e.g., paramedics) to 57.1% among dental nurses. This difference was statistically significant (Fisher's exact test, simulated *p* = 0.025).

A large proportion of cosmetic BoNT practitioners did not provide remedial support when complications arose, ranging from 40.7% in relation to beauticians to 19.1% when the treatment was carried out by medical doctors. Such disparity likely reflects differences in provider training. For example, doctors receive extensive medical education (e.g., pharmacology, anatomy) and significant hands‐on clinical experience including injection technique, whereas beauticians often qualify via a brief course. Moreover, beauticians lack authority to prescribe needed medication and competence to manage adverse reactions.

The referral rate to NHS A&E varied widely by practitioner type from 0% in dentist‐led treatments to 57.1% in those by dental nurses, underscoring inconsistent management pathways and an undue shift of burden onto public healthcare. This data suggests that many patients with complications are referred to NHS A&E rather than treated by the original provider. NHS costs for A&E attendance range from £114 to £563 per case thus such referrals to A&E may equate to a substantial economic burden on the NHS. It is possible that a substantial proportion of these attendances arise when non‐prescribers default to NHS A&E care. This suggests that restricting BoNT provision to senior prescribers with the requisite medical training to resolve adverse events within the private sector may prevent the unnecessary transfer of risk and cost to the NHS.

Our findings show that many providers abdicate responsibility for complications, leaving patients to seek help in A&E departments. This not only increases patient risk but imposes an avoidable cost burden on the NHS. Regulatory reform should require all cosmetic BoNT to be administered only by adequately trained, independent prescribers, capable of managing adverse events. Enforcing such standards, along with clear in‐house complication protocols, would improve patient safety and prevent the default transfer of private‐sector complications onto public services.

## Author Contributions

Writing – original draft: Lee Smith and Roshan Ravindran. Writing – review and editing: Jose Francisco Lopez‐Gil, Masoud Rahmati, and Julia Gawronska. All authors have read and agreed to the published version of the manuscript.

## Ethics Statement

Ethical approval was granted in December 2024 by the Anglia Ruskin University Research Ethics Panel (Ethics ID: ETH2425‐1930).

## Consent

Participants provided informed consent electronically prior to beginning the online survey. All responses were anonymous, and participation was fully voluntary.

## Conflicts of Interest

The authors declare no conflicts of interest.

## Data Availability

The data that support the findings of this study are available on request from the corresponding author. The data are not publicly available due to privacy or ethical restrictions.
